# The Multifunctional Fungal Ergosterol

**DOI:** 10.1128/mBio.01755-18

**Published:** 2018-09-18

**Authors:** Marcio L. Rodrigues

**Affiliations:** a Instituto Carlos Chagas, Fundação Oswaldo Cruz (Fiocruz), Curitiba, Brazil; b Instituto de Microbiologia Paulo de Góes (IMPG), Universidade Federal do Rio de Janeiro, Rio de Janeiro, Brazil

**Keywords:** *Candida albicans*, *Saccharomyces cerevisiae*, antifungal agents, ergosterol, fungi, immunity

## Abstract

The roles of ergosterol in the regulation of membrane fluidity and structure, as well its role as a target for the activity of antifungals, have been known for decades. Two recent studies, however, demonstrated that ergosterol is an immunologically active lipid that induces pyroptosis and that virtually all steps of its biosynthetic process are potential drug targets. In combination, those two reports strongly indicate that the biological relevance of ergosterol is broader than the current literature suggests.

## Abstract (precis)

The roles of ergosterol in the regulation of membrane fluidity and structure, as well its role as a target for the activity of antifungals, have been known for decades. Two recent studies, however, demonstrated that ergosterol is an immunologically active lipid that induces pyroptosis and that virtually all steps of its biosynthetic process are potential drug targets.

## COMMENTARY

Ergosterol, a 5,7-diene oxysterol, is the most abundant sterol in fungal cell membranes, where it regulates permeability and fluidity (1). Because of its crucial functions, unique structural properties, and particular biosynthetic steps, ergosterol is the target of the majority of clinically available antifungals (2). Ergosterol and other sterols might also participate in interkingdom interactions. In plants, ergosterol represents a microbe-associated molecular pattern recognized as an immunologically active “non-self-antigen” (3). Glycosylated sterols have been linked to immunological protection of the host in an animal model of fungal disease (4). Those past discoveries show that fungal sterols unquestionably play essential roles in already known biological processes. However, recent studies from the White, Krysan, and Wellington laboratories suggest that scientists are still uncovering the biological relevance of ergosterol (5, 6).

## ERGOSTEROL AS AN IMMUNOACTIVE FUNGAL MOLECULE

Inflammasome-mediated activation results in a proinflammatory form of programmed cell death known as pyroptosis (7). As reviewed by Man and colleagues (8), pyroptosis is a form of necrotic and inflammatory programmed cell death induced by inflammatory caspases. The caspases required for activation of pyroptosis include human and mouse caspase-1, human caspase-4 and caspase-5, and mouse caspase-11 (8). Caspases mediate cleavage and activation of the pore-forming effector protein gasdermin D. Pore-mediated cell rupture results in the extracellular release of proinflammatory cytokines and alarmins and in danger-associated molecular patterns, which participate in the control of bacterial, viral, fungal, and protozoan infections (7, 8). Results obtained from experimental models by the use of mice deficient in caspase-1 and/or caspase-11 and infected with Aspergillus fumigatus, Paracoccidioides brasiliensis, or Candida albicans have suggested that organ damage, increased fungal dissemination, and reduced host survival might be related to pyroptosis (9–11). Until recently, however, yeast-triggered pyroptosis has been observed only *in vitro*. In their recent study, Koselny and colleagues showed that pyroptosis occurs in the initial stages of murine kidney infection (5).

A previous study demonstrated that C. albicans-induced macrophage pyroptosis is driven by fungal cell-wall remodeling and exposure of glycosylated proteins in response to the macrophage phagosome (12). In the study by Koselny and colleagues, cell surface localization and/or total levels of ergosterol correlated with the ability of S. cerevisiae, C. albicans, and C. neoformans to trigger pyroptosis (5). By using a filamentous strain of S. cerevisiae (Σ1278b) that induces inflammasome activation and a nonfilamentous strain (S288c) that does not, they identified a set of genes involved in ergosterol and membrane homeostasis as candidates to participate in the induction of pyroptosis-related macrophage lysis. Several genes required for macrophage lysis were also required for filamentation (5). This suggests that fungal ergosterol may be the trigger for this mammalian cell process.

Such a relationship between ergosterol and pyroptosis has been implied by previous reports. The key transcriptional regulator of ergosterol biosynthesis, Upc2, was required for C. albicans-induced pyroptosis (9), and functional deactivation of Upc2 function led to a reduced amount of cellular ergosterol (9, 13). In the work from Koselny and colleagues, the first experimental evidence that ergosterol itself directly induces pyroptosis was its increased concentration in pyroptosis-inducing hyphal cells compared to yeast cells (5). This was elegantly confirmed through the use of ergosterol-containing and ergosterol-free liposomes in a macrophage pyroptosis model (5). Only the ergosterol-containing liposomes were able to induce pyroptosis-mediated macrophage lysis. These results strongly suggest that there are mechanisms of ergosterol recognition by macrophages.

One concern about the findings summarized above is related to the fact that ergosterol is a typical plasma membrane component and therefore is not accessible to recognition by macrophage surface receptors. However, a number of C. albicans cell wall mannoproteins and related molecules have been demonstrated to bind and transport extracellular sterols (5), suggesting external distribution. In fact, ergosterol is a major lipid component of fungal extracellular vesicles (14), which are the main vehicles of trans-cell wall transport in fungi (15). The passage of ergosterol-containing vesicles through the cell wall implies that this sterol is a transitory cell wall component (16), which might facilitate recognition by immune cells. In addition, ergosterol-containing vesicles released extracellularly by C. albicans promote macrophage stimulation (17). Therefore, a number of regular physiological processes support ergosterol export and consequent participation in immunological events. In this scenario, Koselny and colleagues (5) were the first to demonstrate that fungal ergosterol is immunologically active, which opens several avenues of investigation on how the host responds to sterols and fungal lipids in general.

## ERGOSTEROL BIOSYNTHESIS PATHWAYS PROVIDE MULTIPLE TARGETS FOR ANTIFUNGAL DEVELOPMENT

The need for novel antifungals is unquestionable. Recent estimates report 1.6 million human deaths each year due to systemic mycoses (18), and the currently available therapeutic options are unaffordable, toxic, or inefficient (19). Ergosterol biosynthesis is still the most important cellular pathway targeted by antifungal compounds (2).

Ergosterol is synthesized in the endoplasmic reticulum through the sequential activity of 25 different enzymes (2). Upc2, the transcription factor required for pyroptosis in the C. albicans model, is directly related to the ergosterol biosynthesis pathway. This transcriptional regulator senses the intracellular levels of sterols, which results in the activation of genes required for sterol uptake and biosynthesis (13). In the light of the findings reported by Koselny and colleagues (5) and the literature mentioned above, Upc2 is a central transcription factor regulating both fungal physiology and immunopathogenesis.

The currently available antifungal classes interfering with ergosterol synthesis affect the products of the *ERG11* gene (14α-demethylase; azoles), the *ERG1* gene (squalene epoxidase; allylamines), and the *ERG2* gene (sterol C-8 isomerase; morpholines). The potential of the 22 remaining genes required for ergosterol biosynthesis as antifungal targets remains to be explored. Most genes required for ergosterol synthesis are essential, which imposes difficulties with respect to the characterization of antifungal targets. In this scenario of great complexity, Bhattacharya and colleagues (6) overexpressed each of the 25 genes encoding the enzymes required for ergosterol biosynthesis in S. cerevisiae and characterized the phenotypic traits of each overexpressing strain in the presence of different stress agents. The impact of gene overexpression was remarkable. Strains with increased expression of *HMG1*, *ERG9*, *ERG1, NCP1, ERG25, ERG27, ERG28, ERG6*, and *ERG2*, for instance, had decreased growth rates in rich medium and had their growth completely inhibited under conditions of iron deprivation. Calcium availability was also linked to ergosterol metabolism, as concluded from the lack of growth of 22 of the 25 strains in low calcium concentrations. Increased salt osmolarity also negatively affected the growth of 19 of the 25 strains, while cell wall perturbation with Congo red produced growth defects in 10 strains. These results efficiently illustrate the impact of ergosterol metabolism on fungal physiology and, consequently, reinforce the idea of the multiplicity of potential antifungal targets related to ergosterol biosynthesis. This view is supported by a global analysis of the findings reported by Bhattacharya and colleagues: overexpression of two genes (*ERG2* and *ERG6*) had an effect on the activities of all stress agents, while overexpression of all 25 genes had an effect on the activity of at least one agent (6).

Overexpression or viable deletion of ergosterol biosynthesis genes differently impacted the susceptibility of S. cerevisiae to fluconazole, fenpropimorph, lovastatin, nystatin, amphotericin B, or terbinafine (all affecting fungal ergosterol) (6). In particular, exposure of the overexpressing strains to lovastatin, terbinafine, fluconazole, or fenpropimorph revealed that the previously known primary drug targets were not the only genes involved in the antifungal activity. Altogether, these results efficiently illustrate the view that the targets for antifungal development related to the ergosterol synthesis pathway are much more numerous than is understood in current practice.

## PERSPECTIVES

**FIG 1:**
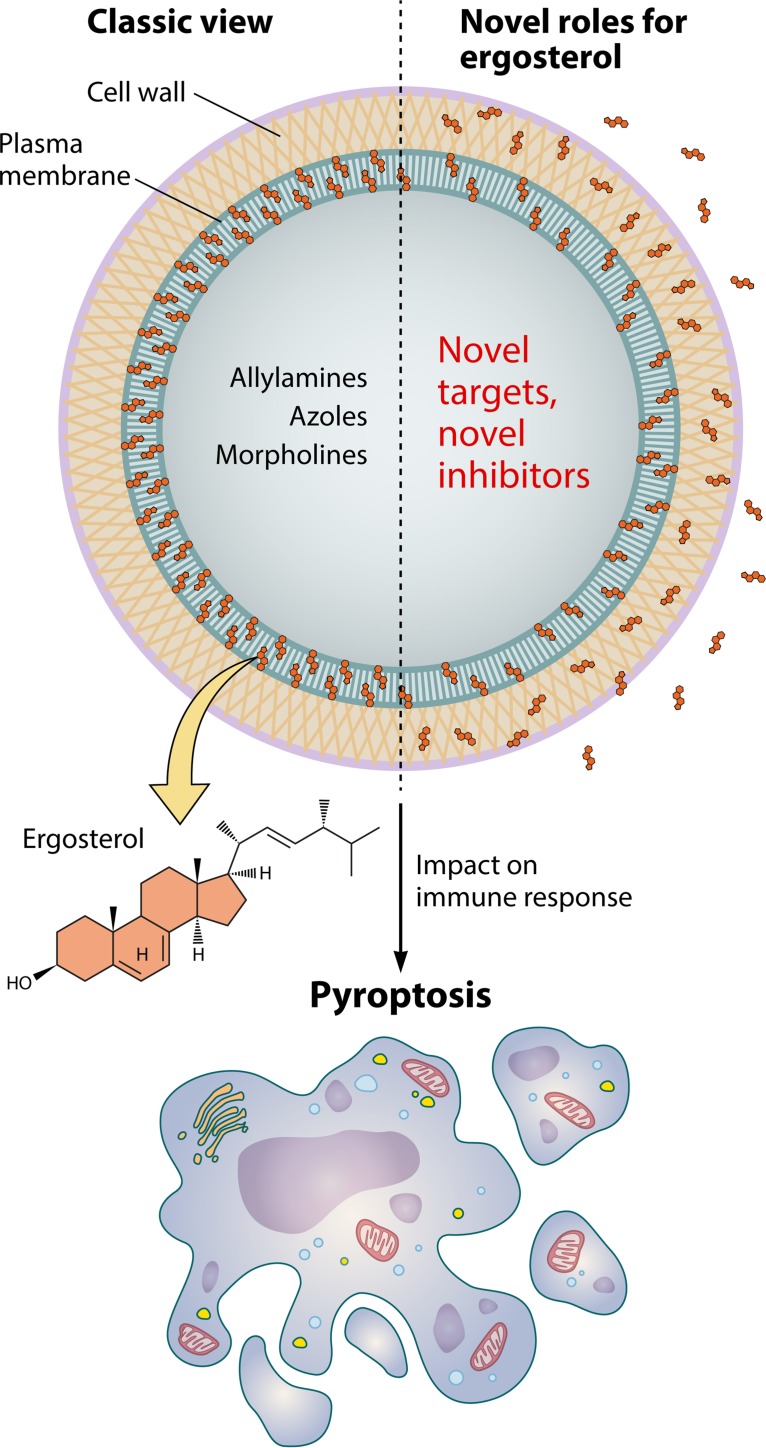
The multiple roles of ergosterol in fungal cells. Ergosterol is a regulator of the fluidity of fungal membranes (left panel, classic view). On the basis of the structural differences between ergosterol and mammalian cholesterol, the antifungals allylamines, azoles, and morpholines can selectively inhibit 3 of the 25 enzymes required for biosynthesis of the fungal sterol. The studies by Koselny et al. (5) and Bhattacharya et al. (6) demonstrate novel roles for fungal ergosterol (right panel). Their observations imply that additional antifungal classes have the potential to target ergosterol biosynthesis and that this lipid interacts with the immune system, inducing pyroptosis. The immunological functions of ergosterol are compatible with the notion that this fungal sterol, rather than being limited to the plasma membrane, has a broad distribution in the fungal cell.

The surface of fungal cells has been long recognized as a static molecular complex with exclusive structural functions. This classic view changed radically during recent decades after the demonstration that surface polysaccharides with well-described structural roles, including glucans, chitin, and mannan, were identified as key regulators of immunological activity (reviewed in reference 20). Fungal lipids, which were thought to be plasma membrane components exclusively, were demonstrated to be surface and extracellular components with key roles in fungal virulence (21). These seminal observations and the report by Koselny and colleagues (5) clearly demonstrate that, rather than being a rigid structure with structural roles, the fungal surface is a highly dynamic molecular complex with great potential to stimulate the host’s immune response (Fig. 1). In addition, the multiplicity of composition and functions of the fungal cell surface agrees with the notion that a number of selective antifungal targets remain to be discovered, as demonstrated by Bhattacharya and coworkers (6). The studies by Koselny and Bhattacharya and their colleagues (5, 6) open up new views on how the host interacts with fungal cells and how new possibilities of pharmacological interference in this process can be explored.
